# High Recurrence Rates of Hyperextension After Total Knee Arthroplasty in Asian Patients With Preoperative Knee Hyperextension: A Prospective Observational Study

**DOI:** 10.7759/cureus.43305

**Published:** 2023-08-10

**Authors:** Rajshekhar K Thippanna, Malhar N Kumar, Adarsh Krishna K Bhat

**Affiliations:** 1 Orthopaedic Surgery, Apollo Hospitals, Bangalore, IND; 2 Orthopaedics, East Point College of Medical Sciences, Bangalore, IND

**Keywords:** osteoarthritis, recurrence rate, hyperextension, genu recurvatum, knee arthroplasty

## Abstract

Introduction: Genu recurvatum is a well-known problem in total knee arthroplasty (TKA) in patients with and without neuromuscular disorders. Hyperextension of the knee joint does not reoccur significantly following adequate correction during TKA is the generally accepted notion. The literature regarding the reoccurrence of genu recurvatum in patients after TKA with preoperative genu recurvatum is scarce. The current study is an intermediate-range follow-up study to assess the pre- and postoperative sagittal plane profiles of Asian patients with genu recurvatum who underwent TKA. Changes in the sagittal profile in the immediate postoperative period were compared with the sagittal plane profile during the follow-up to the time of the final follow-up.

Materials and methods: This study was a prospective observational study of 21 patients (35 knees) with preoperative hyperextension of the affected knee who underwent total knee arthroplasty between July 2014 and September 2018, in our centre. The inclusion criteria were patients with primary osteoarthritis of the knee with recurvatum deformity ≥5° as measured preoperatively on a standing lateral radiograph. The exclusion criteria were neuropathic joints, post-traumatic arthritis, inflammatory arthritis, arthritis-associated neuromuscular disorders and revision procedures. The preoperative patients were divided into two groups: those with hyperextension of ≤10° and those with hyperextension of >10°. Radiographic measurements were done using the DICOM software (Kriens, Switzerland). The mean follow-up was 4.7 years (range: 3.6 to 7.6 years), and the minimum follow-up period was 3.6 years. No patients were lost to follow-up. All patients were evaluated clinically pre- and postoperatively using the Knee Society score. The knee range of movement and the coronal and sagittal profiles were recorded using standing radiographs. Statistical evaluation was done using the Chi-square test and the Wilcoxon signed-rank tests (SPSS version 17, Chicago, IL SPSS Inc, 2008).

Results: Twenty-one patients (35 knees) with preoperative knee hyperextension underwent total knee arthroplasty with the mean age of patients being 59.38 years and the mean BMI of 32.28. The mean preoperative hyperextension was -10.1° (range: -5° to -26°). Early postoperative sagittal alignment (mean) was +4.5° (3° to 10°), and the mean sagittal alignment at final follow-up was -10.9° (-5° to -15°) (positive values indicate residual knee flexion, and negative values indicate hyperextension). There was no significant difference in the preoperative sagittal profiles of patients with BMI <30 and ≥30 (p=0.43). There was no statistically significant difference (p=0.19) between those with hyperextension of ≤10° and those with hyperextension of >10°.

Conclusion: Till now, none of the patients have complained of symptoms related to hyperextension, although the rate of recurrence of hyperextension is high. Long-term follow-up is essential in patients with recurvatum deformity who have undergone TKA since delayed recurrence of hyperextension is possible despite adequate intraoperative correction of the deformity. Accurate preoperative prediction about the magnitude of postoperative deformity is not feasible. It is essential to counsel patients preoperatively that hyperextension may recur even after exercising sufficient care in the operative procedure to minimize its occurrence.

## Introduction

Genu recurvatum is a well-known problem in total knee arthroplasty (TKA) in patients with [1-3] and without [4-9] neuromuscular disorders. Earlier studies have reported a prevalence between 1% and 3.9% of preoperative genu recurvatum in knees with osteoarthritis that required TKA [4-7]. It has been claimed that hyperextension does not recur significantly following adequate correction during TKA [4-7]. The mean follow-up period of these studies ranged from 2 years to 4.5 years [1,4-7]. The only study on recurvatum in the Asian population published so far has a short mean follow-up of around 2.2 years [7]. The current study is an intermediate-range follow-up study to assess the pre- and postoperative sagittal plane profiles of Asian patients with genu recurvatum who underwent TKA. Changes in the sagittal profile in the immediate postoperative period were compared with the sagittal plane profile during the follow-up to the time of the final follow-up.

## Materials and methods

This was a prospective follow-up study of 21 patients (35 knees) with preoperative hyperextension of the affected knee who had undergone TKA between July 2014 and September 2018. A total of 1,340 patients underwent TKA in our centre during that period. The inclusion criteria were patients with primary osteoarthritis of the knee with recurvatum deformity ≥5° as measured preoperatively on a standing lateral radiograph. The exclusion criteria were neuropathic joints, post-traumatic arthritis, inflammatory arthritis, arthritis-associated neuromuscular disorders and revision procedures. In this way, we identified 21 patients (five men and 16 women) with primary osteoarthritis of the knee with recurvatum deformity giving a prevalence of recurvatum of 1.6% in osteoarthritic patients undergoing TKA in our institution. Seven patients had unilateral involvement, and 14 patients had bilateral involvement.

Patients were evaluated according to the Knee Society clinical and radiographic evaluation systems before surgery and at each follow-up. Standing anteroposterior (AP) and lateral radiographs were taken before surgery and at each follow-up. Patients were included in this study if their preoperative examination revealed at least 5° of knee hyperextension.

All patients underwent TKA using a cemented, posterior cruciate-substituting implant along with patellar resurfacing (PFC Sigma®; DePuy, Warsaw, Indiana). The deformity in the sagittal and coronal planes was assessed intraoperatively after exposure of the joint and excision of the cruciate ligaments and menisci and prior to any capsuloligamentous release. The tibia was resected first, and the amount of bony resection of the femur was performed depending upon the severity of the deformity: the greater the recurvatum, the less the resection. Particular care was taken to avoid the posterior slope in the tibial cut (a neutral tibial cut was used). Similarly, care was taken to avoid the flexed position of the femoral component. Using the gap-balancing technique (spacer blocks), the amount of soft-tissue release was determined by the degree of soft-tissue tightness. Care was taken to perform no capsular release posteriorly. Limb alignment and gap balancing were re-checked using trial components. Definitive components were implanted with cement if the coronal and sagittal alignments with the trials were satisfactory. It was ensured that there was no residual recurvatum prior to the definitive implantation of the TKA components.

Postoperatively, patients were mobilised full weight-bearing, with active knee flexion commenced on the first postoperative day. Clinical and radiographic evaluations were done at each follow-up commencing four weeks after surgery and then at three months, six months and annually during follow-up visits. Radiographic measurements were done using the DICOM software (Kriens, Switzerland). The mean follow-up was 4.7 years (range: 3.6 to 7.6 years), and the minimum follow-up period was 3.6 years. No patient was lost to follow-up. All patients were evaluated clinically pre- and postoperatively using the Knee Society score. The knee range of movement and the coronal and sagittal profiles were recorded using standing radiographs. Statistical evaluation was done using the Chi-square test and the Wilcoxon signed-rank tests (SPSS version 17, Chicago, IL SPSS Inc, 2008).

## Results

**Figure 1:**
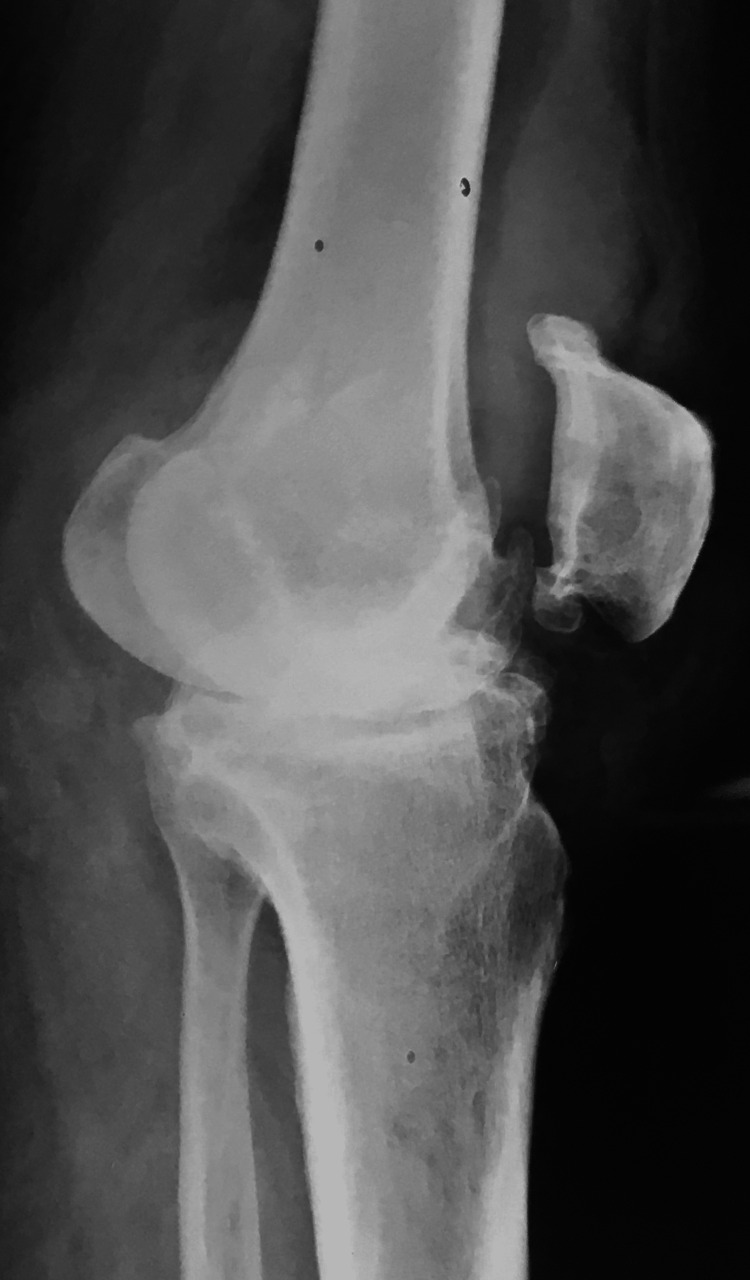
Preoperative standing lateral X-ray view For the X-ray, consent and permission were obtained from the patient.

**Figure 2:**
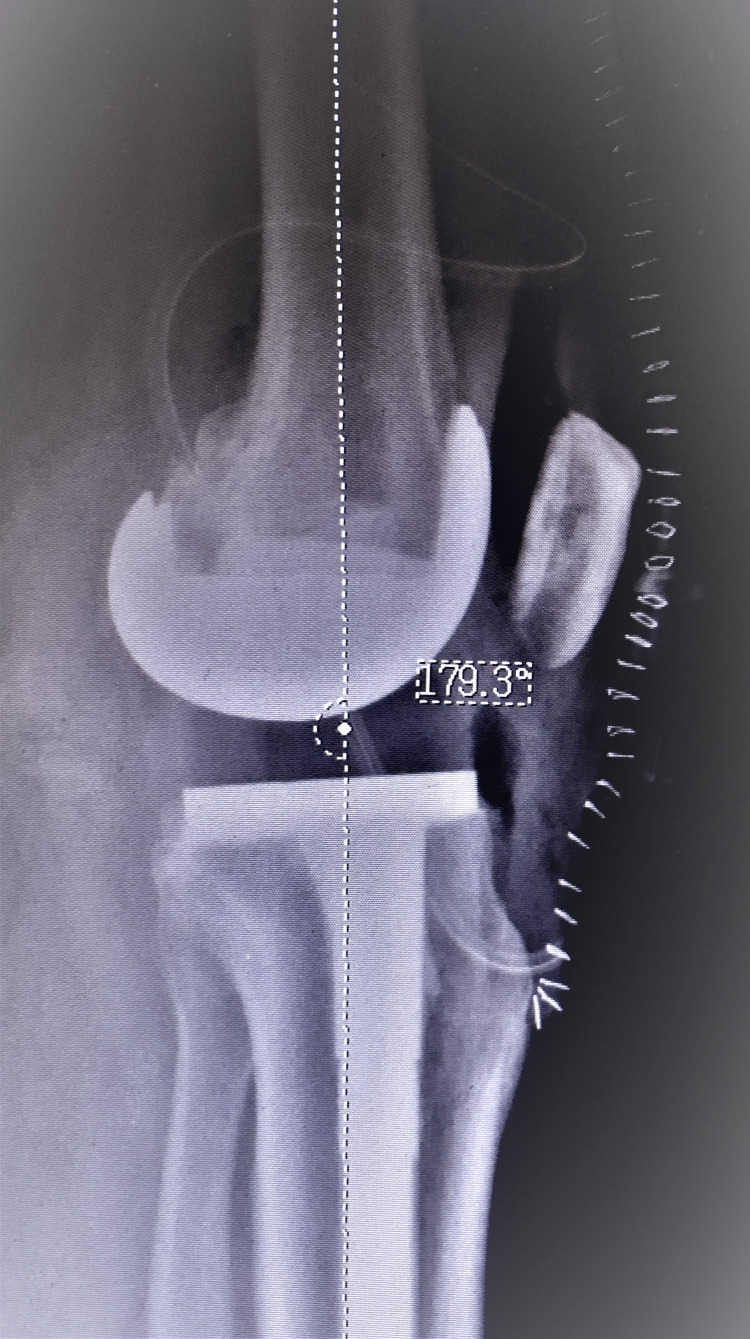
Immediate postoperative lateral view X-ray For the X-ray, consent and permission were obtained from the patient.

**Figure 3:**
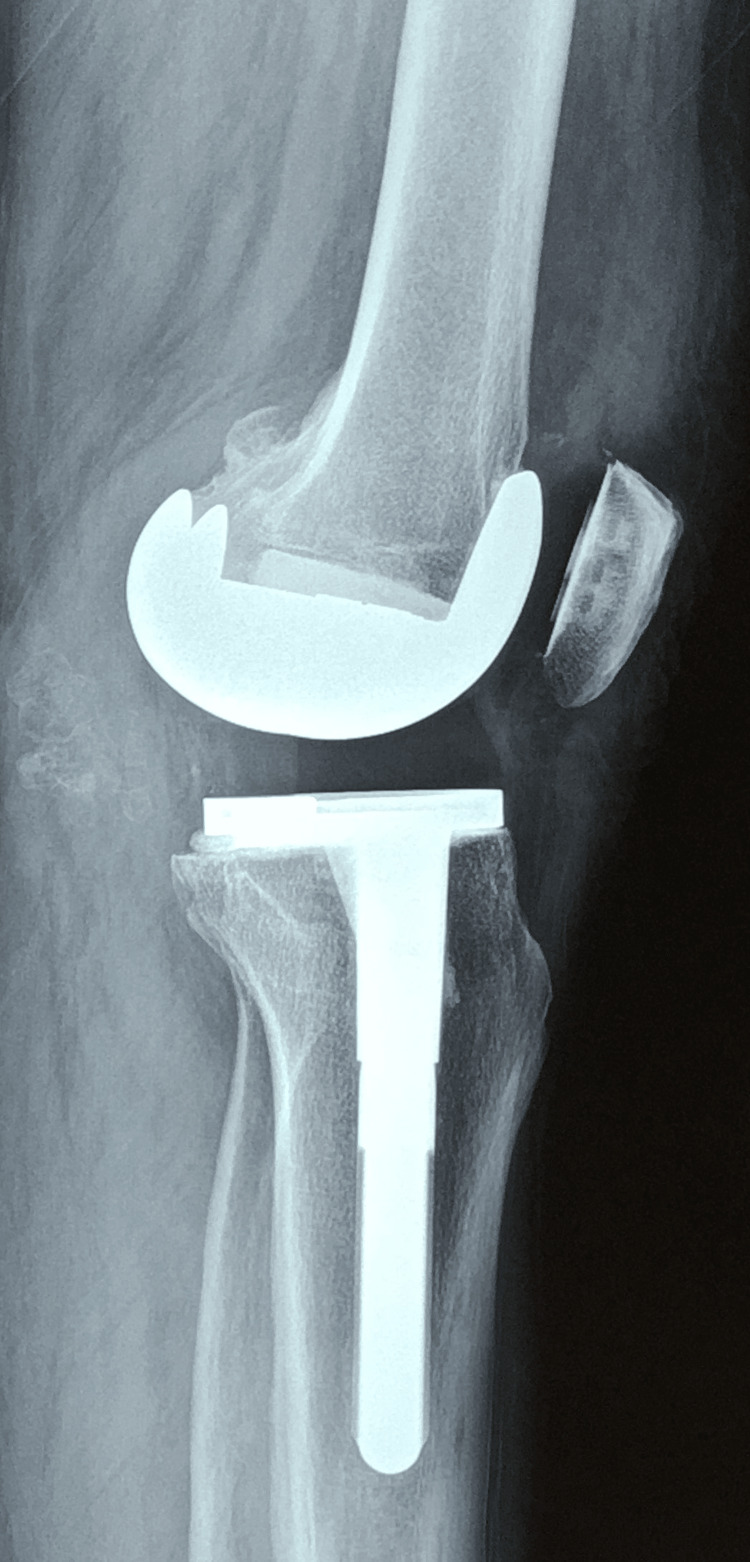
Two-year follow-up, lateral view X-ray For the X-ray, consent and permission were obtained from the patient.

The mean age of patients was 59.38 years (SD ± 9.9). The mean BMI was 32.28 (SD ± 3.6). The mean preoperative hyperextension was -10.1° (range: -5° to -26°). Early postoperative sagittal alignment (mean) was +4.5° (3° to 10°), and the mean sagittal alignment at the final follow-up was -10.9° (-5° to -15°) (positive values indicate residual knee flexion, and negative values indicate hyperextension). Details of individual patients are shown in Table 1. There was a statistically significant difference between the preoperative and immediate postoperative sagittal plane profiles (p<0.001). This showed that recurvatum had been corrected well during TKA (knees were kept either neutral or in mild flexion at the end of the operation). There was a statistically significant difference (p<0.001) between the sagittal profiles in the immediate postoperative and final follow-up periods. This showed that the recurvatum deformity had recurred in most patients at the final follow-up (Figure 3). Lateral X-ray views of the trends of hyperextension over time are shown in Figures 1-3.

**Table 1:** Clinical data including BMI and knee extension magnitudes

No.	Age	Sex	BMI	Knee extension (preoperative)	Knee extension (immediate postoperative)	Knee extension (final follow-up)
1	54	F	38	-10	+5	-16
2	63	F	38	-5	+7	-12
3	51	F	37	-5	0	-5
				-10	0	-15
4	68	M	36	-14	+6	-10
5	50	F	38	-26	+10	-15
6	52	F	28	-16	+5	-19
				-19	+5	-21
7	69	M	36	-10	+5	-7
				-10	+5	-10
8	56	F	32	-9	+10	-5
9	55	F	30	-8	+5	-12
				-5	+5	-10
10	58	F	36	-15	0	-10
				-10	+5	-10
11	58	F	30	-18	0	-18
				-10	+5	-20
12	68	M	30	-10	0	-10
				-12	+5	-10
13	56	F	30	-5	0	-10
				-9	+5	-8
14	58	M	32	-15	+6	-15
15	58	F	29	-5	+7	-5
				-5	+3	-10
16	67	F	30	-5	0	-10
				-8	+5	-7
17	48	F	28	-5	+3	-10
				-10	+5	-10
18	66	M	30	-15	+5	-10
19	69	F	30	-5	+7	-10
				-5	+5	-8
20	71	F	32	-8	+5	-12
				-5	+5	-8
21	52	F	28	-10	+8	-6
				-15	+5	-10

There was no significant difference in the preoperative sagittal profiles of patients with BMI <30 and ≥30 (p=0.43). We divided the preoperative patients into two groups: those with hyperextension of ≤10° and those with hyperextension of >10°. We assessed whether there was a higher incidence of postoperative hyperextension of higher magnitude in those with preoperative hyperextension of >10°. However, there was no statistically significant difference (p=0.19) between the two groups.

At the final follow-up, there was a significant improvement in the mean Knee Society Score (KSS). The mean preoperative KSS pain score was 30.1 (SD: 8.7), and the functional score was 26.4 (SD: 12.3). Postoperative pain score was 90.5 (SD: -7.7), and the functional score was 92 (SD: -8.5). The difference in the pre- and postoperative KSS was significant in pain and function scores (p<0.0001). The mean range of flexion significantly improved from 72° (60° to 105°) preoperatively to 110° (105° to 120°) at the final follow-up (p<0.01). Till now, none of the patients have complained of symptoms related to hyperextension.

## Discussion

Genu recurvatum in association with osteoarthritis is not an uncommon occurrence. Few studies around the millennium reported a 1% prevalence of recurvatum in patients with osteoarthritis awaiting TKA. Later, in 2012, Mullaji et al. identified a higher prevalence (3.9%) of preoperative genu recurvatum in Asian patients [7]. The difference in the prevalence rates of genu recurvatum between previous literature and Mullaji et al. may be attributable to the assessment of knees after anaesthesia, use of navigation and ethnic differences among patients [7]. The high rate of recurrence of hyperextension following TKA is well documented in patients with neuromuscular disorders. In a study reviewing the results of TKA in 16 knees affected with poliomyelitis, Giori and Lewallen [1] reported that the recurrence of instability and progressive functional deterioration is possible in all knees affected with such a neuromuscular condition. However, the recurrence rates of hyperextension have been said to be low following TKA in patients without underlying neuromuscular conditions. Insall and Haas [8] observed that a knee that does not hyperextend at the conclusion of TKA would not develop recurvatum later except in patients who lack muscle control. Similarly, Schurman et al. [9] and Meding et al. [5,6] reported that in knee replacements in patients with genu recurvatum without major neuromuscular diseases, significant increases in extension did not occur after hospital discharge. However, our experience has been different, and the recurrence of preoperative recurvatum was seen in our series despite adequate intraoperative correction of the deformity. The Knee Society (KS) scores in our patients did not show any deterioration despite the recurrence of hyperextension deformity. Longer-term studies are necessary to see whether hyperextension becomes symptomatic.

In our study, there was no significant association between the magnitude of preoperative hyperextension and obesity (defined as BMI≥30). Giori and Lewallen [1] reported a correlation between diminished quadriceps strength and the recurrence of hyperextension along with less pain relief in their patients with neuromuscular involvement. Our patients had no neuromuscular weakness, and there was no weakness in the quadriceps.

**Figure 4:**
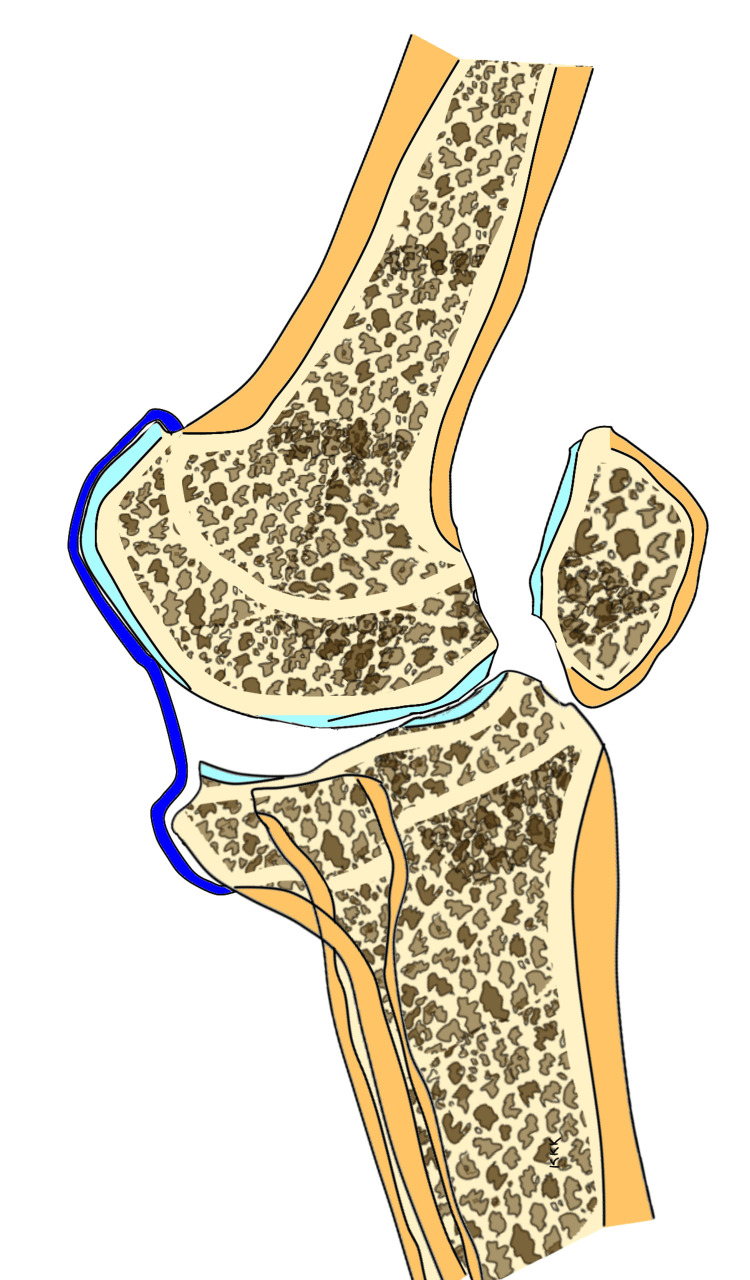
Preoperative hyperextended knee with stretched-out posterior capsule The sketch is the work of the author.

**Figure 5:**
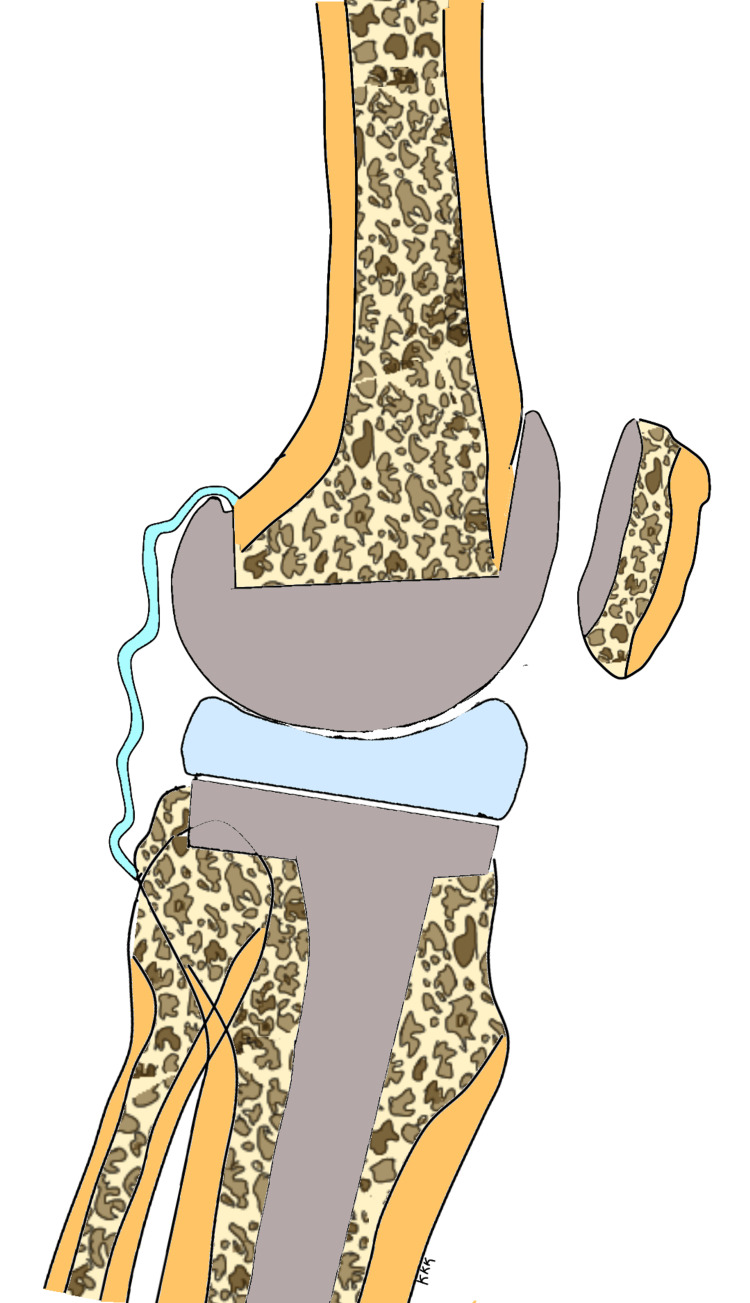
Early postoperative status with adequate correction of deformity and a lax and redundant posterior capsule The sketch is the work of the author.

**Figure 6:**
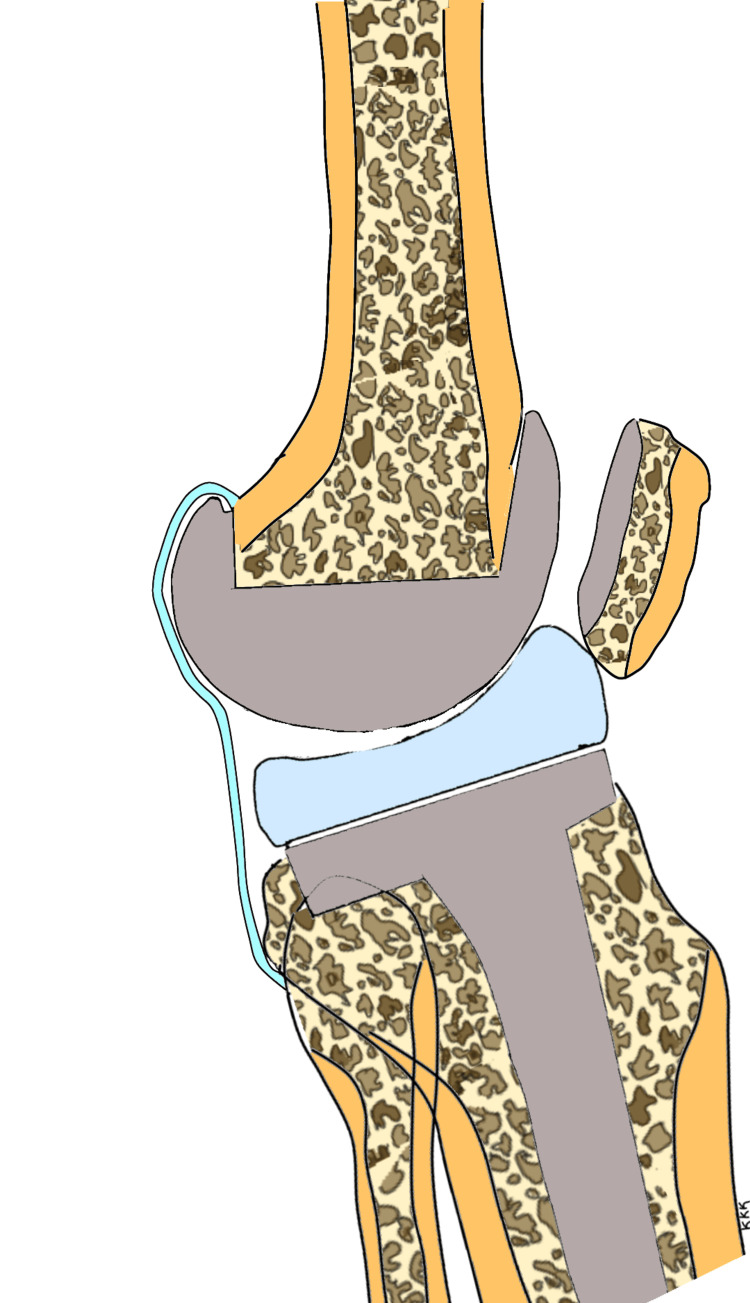
Late postoperative phase showing the recurrence of deformity and stretching out of the posterior capsule (as in the preoperative phase) The sketch is the work of the author.

Patients with preoperative genu recurvatum need to be followed up for longer durations following TKA to detect recurrence of hyperextension. The recurrence of hyperextension deformity is perhaps a function of time, i.e. duration of postoperative follow-up and the elasticity of the connective tissues. The longer the follow-up, the higher the likelihood of recurrence of hyperextension in these patients. The effect of the capsular and ligamentous elasticity does not end with intraoperative gap balancing but continues into the postoperative period and may produce gradual, undesirable changes in the alignment following TKA. The posterior capsule is stretched out in the preoperative phase in patients with hyperextension as shown in Figure 4. This situation reverses following correction of the deformity, and the posterior capsule becomes lax, but the posterior dead space remains since posterior capsular plication is not usually performed at the time of TKA as shown in Figure 5. This provides the potential for the posterior capsule to stretch out once again in the delayed postoperative phase and cause the recurrence of the deformity as shown in Figure 6.

Banks et al. [10] found in fluoroscopic studies that nearly 40% of knees with TKA hyperextend during activities of daily living. In many cases of TKA in their study, the components were aligned in around 10° of hyperextension with respect to the sagittal mechanical axis. These factors may contribute to the postoperative recurrence of the hyperextension deformity in patients with preoperative hyperextension.

Mortazavi et al. [11] found that genu recurvatum can occur in patients with ligamentous hyperlaxity who undergo TKA using a single radius design. Isolated polyethylene exchange could be considered in treating this uncommon complication. Using thicker polyethylene liners at the time of primary TKA in patients with ligamentous hyperlaxity might prevent this complication. Koo et al. [12] in a comparative study of fixed flexion deformity vs genu recurvatum after total knee arthroplasty concluded that it is better to err on the side of fixed flexion deformity if neutral alignment cannot be achieved.

Siddiqui et al. [13] in a study to assess the function and quality of life in patients with recurvatum deformity after TKA found that patients with hyperextension at six months are 6.5 times more likely to have recurvatum at two years vs those with no hyperextension at six months, and postoperative recurvatum of more than 5° significantly impacts function and quality of life of patients. 

In primary TKA for osteoarthritic knees with mild deformities (not more than 10° of recurvatum), the standard semi-constrained fixed-bearing prosthesis is probably sufficient along with good correction of deformity and minimal bone resection. Care should be taken to avoid the hyperextended component position of the components during TKA. It is essential to keep the tibial cut neutral (avoid posterior sloping tibial cut) and maintain the neutral position of the femoral component (avoid flexion of the femoral component). Particular care should be taken to avoid the flexed position of the femoral component, especially in patients whose preoperative radiographs show distal femoral bowing. Hinged (fully constrained) TKA may be the best of currently available options in cases with large recurvatum deformities. Erceg and Rakić reported a case of recurring post-TKA genu recurvatum (55°) presenting upon weight-bearing in a 73-year-old female who underwent three revision surgeries until a Rotating Hinged Knee (RHK) prosthesis could treat the condition [14]. Wong reported a 77-year-old female presenting with increasing genu recurvatum (45°) after TKA, which was also finally treated by RHK. However, the authors believed that early intervention might have obviated the need for a highly constrained prosthesis [15]. An RHK prosthesis has been regarded as the preferred technique for correcting post-TKA genu recurvatum by Cottino et al. [16]. The clinical performance of hinged prostheses has improved with modern designs such as the rotating hinge, but implant survival rates are still below par in comparison with the standard semi-constrained TKA.

Limitations of the study include a small number of cases as hyperextension does not recur significantly following adequate correction during TKA [4-7]. The mean follow-up period in this study is 4.7 years, which though higher than most other published studies on this topic [1-4,7], is another limiting factor and warrants the need for further follow-up and assessment.

## Conclusions

Long-term follow-up is essential in patients with recurvatum deformity who have undergone TKA since delayed recurrence of hyperextension is possible despite adequate intraoperative correction of the deformity. Accurate preoperative prediction about the magnitude of postoperative deformity is not feasible. Prolonged postoperative follow-up for at least 10 years duration is essential in these patients. It is essential to avoid hyperextended component positioning while performing TKA on knees with preoperative hyperextension. Clinical observation of the absence of recurvatum is not sufficient in these patients. It should be measured on standing lateral radiographs. There can be hyperextension thrust even in knees with clinically straight knees without hyperextension deformity if the component alignment is biased towards extension (posterior slope of the tibial component and flexed position of the femoral component). It is essential to counsel patients preoperatively that hyperextension may recur even after exercising sufficient care in the operative procedure to minimize its occurrence.
